# Supplementary material for: Development of Mucosal PNAd+ and MAdCAM-1+ Venules during Disease Course in Ulcerative Colitis
Source: Cells. 2020 Apr 6;9(4):891. doi: 10.3390/cells9040891 (PMC7226824; doi:10.3390/cells9040891)
Supplement: Supplementary file 1 [file cells-09-00891-s001.pdf]

Supplementary table S1. Patient characteristics at follow-up endoscopy.

	All UC patients n=378	Non-inflamed UC at follow-up n=93	Inflamed UC at follow-up n= 285	p-value**
**Clinical follow-up time in months**	128 (85-165)	150 (106.5-169.5)	121 (82-159)	0.002
**Time till first exacerbation in months**	25 (10-59)	48 (18-125)	21 (9-49)	0.001
- No exacerbation during follow-up, n (%)	41 (10.8%)	28 (30.1%)	13 (4.6%)*	
- Exacerbation, n (%)	337 (89.2%)	65 (69.9%)	272 (95.4%)	
**Surgery during clinical follow-up**	24 (6.3%)	2 (2.2%)	22 (7.7%)	0.06
**Mayo endoscopic score**				0.001
- Mayo 0	93(24.6%)	93(100%)	-	
- Mayo 1	107 (28.3%)	-	107 (37.5%)	
- Mayo 2	124 (32.8%)	-	124 (43.5%)	
- Mayo 3	54 (14.3%)	-	54 (18.9%)	
**UC localization**				0.001
- Extent				
o E1: Ulcerative proctitis	86 (22.8%)	-	86 (30.2%)	
o E2: Left-sided UC	130 (34.4%)	-	130 (45.6%)	
o E3: Extensive UC	69 (18.3%)	-	69 (24.3%)	
o Remission	93 (24.6%)	93 (100%)	-	
**Histological inflammation Geboes score**				0.001
- ≥0.1 and <3.1	151 (43%)	80 (86%)	71 (25%)	
- ≥3.1	217 (57%)	13 (14%)	214 (75%)	
**Treatment at follow-up endoscopy**				0.002
- None	100 (26.5%)	21 (22.6%)	79 (27.7%)	
- 5-ASA	152 (40.2%)	44 (47.3%)	108 (37.9%)	
- Topical steroids	14 (3.7%)	1 (1.1%)	13 (4.6%)	
- Oral steroids	37 (9.8%)	1 (1.1%)	36 (12.6%)	
- Immunomodulators	40 (10.6%)	16 (17.2%)	24 (8.4%)	
- Anti-TNF	32 (8.5%)	10 (10.8%)	22 (7.7%)	
- Anti-integrins	3 (0.8%)	0 (0%)	3 (1.1%)	
**Highest treatment during follow-up**				0.06
- None	1(0.3%)	0 (0%)	1 (0.4%)	
- 5-ASA	88 (22.7%)	25 (26.9%)	63 (22.1%)	
- Topical steroids	52 (13.4%)	8 (8.6%)	44 (15.4%)	
- Oral steroids	42 (10.8%)	11 (11.8%)	31 (10.9%)	
- Immunomodulators	101 (26%)	25 (26.9%)	76 (26.7%)	
- Anti-TNF	62 (16%)	22 (23.7%)	40 (14.0%)	
- Anti-integrins	9 (2.3%)	0 (0%)	9 (3.2%)	
- Surgery	23 (5.9%)	2 (2.2%)	21 (7.4%)	

Values expressed in n (%) or as median with interquartile range.

*Biopsies were taken during follow-up endoscopy in UC patients who did not reach remission after initial diagnosis. ** Non-inflamed UC compared to inflamed UC patients; HC, Healthy control; UC, ulcerative colitis.

Supplementary table 2. Univariable and multivariable analysis of potential variables correlating with the expression of PNAd⁺ and MAdCAM-1⁺ venules as a proportion of ERG⁺ venules in active disease at follow-up endoscopy (A+B).

A. Follow-up PNAd	*Univariable*			*Multivariable*	
	R^2 (%)	β (SE)	p-value	β (SE)	p-value
**Follow-up PA is the first exacerbation***	3.2	-0.18 (0.005)	**0.08**		NS
**Disease location ****	33.5	-0.58 (0.002)	**0.001**	-0.37 (0.002)	**0.001**
**Histologic disease activity**					
- **Geboes 0**	19.2	0.44 (0.004)	**0.001**		NS
- **Geboes 1**	21.7	0.47 (0.004)	**0.001**		NS
- **Geboes 2A**	19.1	0.44 (0.004)	**0.001**		NS
- **Geboes 2B**	27.4	0.52 (0.003)	**0.001**		NS
- **Geboes 3**	29.2	0.54 (0.005)	**0.001**		NS
- **Geboes 4**	32.0	0.57 (0.005)	**0.001**	0.33 (0.006)	**0.003**
- **Geboes 5**	17.8	0.42 (0.003)	**0.001**		NS

*Yes=1, no=2 ** Proctitis=1, left-sided=2, extensive=3
β=Regressioncoefficient, SE=Standard Error

B. Follow-up MAdCAM-1	*Univariable*			*Multivariable*	
	R^2 (%)	β (SE)	p-value	β (SE)	p-value
**Follow-up PA is the first exacerbation***	5.6	-0.24 (0.012)	**0.02**		NS
**Disease location****	0.1	-0.04 (0.006)	0.71	-	-
**Histologic disease activity**					
- **Geboes 0**	1.0	0.10 (0.011)	0.34	-	-
- **Geboes 1**	4.5	0.21 (0.01)	**0.04**	**0.26 (0.01)**	NS
- **Geboes 2A**	6.9	0.26 (0.01)	**0.01**		**0.01**
- **Geboes 2B**	5.3	0.23 (0.01)	**0.02**		NS
- **Geboes 3**	2.7	0.16 (0.013)	**0.11**		NS
- **Geboes 4**	1.7	0.13 (0.014)	**0.20**		NS
- **Geboes 5**	0.6	0.08 (0.009)	0.47	-	-

*Yes=1, no=2 ** Proctitis=1, left-sided=2, extensive=3
β=Regressioncoefficient, SE=Standard Error

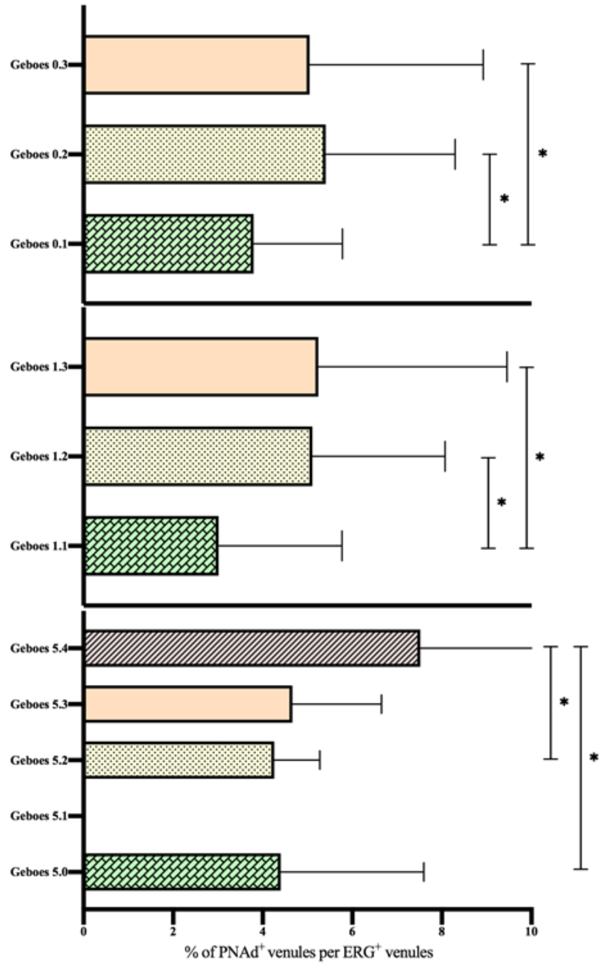

Supplementary Figure 1 . Grades 0 (Architectural changes), 1 (Chronic inflammatory infiltrate) and 5 (The presence of erosions and ulcerations) of the Geboes score for assessment of ulcerative colitis histological disease activity correlate with % of $PNAd^+$ venules per ERG^+ venules. There was no significant association between the Geboes Grades 2 (Eosinophils and neutrophils in the lamina propria),3 (Neutrophils in the epithelium) and 4 (Crypt destruction) and the proportion of $PNAd^+$ venules (not shown in this figure). * p-value < 0.05. ERG, ETS related gene; PNAd, peripheral node addressin; UC, ulcerative coliti
